# The effects of Tai Chi and Baduanjin on breast cancer patients: systematic review and meta-analysis of randomized controlled trials

**DOI:** 10.3389/fonc.2024.1434087

**Published:** 2024-10-28

**Authors:** Yifang Chen, Xinyi Zuo, Yong Tang, Zhimiao Zhou

**Affiliations:** ^1^ Institution of Policy Studies, Lingnan University, Tuen Mun, Hong Kong SAR, China; ^2^ Sociology Department, School of Government, Shenzhen University, Shenzhen, Guangdong, China; ^3^ Shenzhen Senior High School, Shenzhen, China

**Keywords:** Tai Chi and Baduanjin, rest breaks, meditation, relaxation, mindfulness, mental health, breast cancer patients

## Abstract

**Background:**

Tai Chi and Baduanjin are nonpharmacological interventions that are widely applied among cancer patients.

**Objective:**

This meta-analysis aimed to assess the effect of Tai Chi and Baduanjin on breast cancer patients by summarizing and pooling the results of previous studies.

**Methods:**

The PubMed, Embase, Web of Science, Scopus and Cochrane Library and several databases were searched up to December 1, 2023, to identify high-quality RCTs. Relevant terms such as Tai Chi and Baduanjin were used as keywords. Stata 15.0 software and Review Manager (version 5.3; Cochrane Training) were used to screen the studies, extract the data, code the data, and perform the meta-analysis. The mean differences (MDs) and standardized mean differences (SMDs) with 95% CIs were used to calculate continuous variables. The Cochrane risk of bias assessment tool was used to evaluate the risk of bias. The PICOS framework was used to develop the following eligibility criteria: (i) population - breast cancer patients; (ii) intervention - Tai Chi and Baduanjin intervention; (iii) comparison - Tai Chi and Baduanjin group and different intervention (e.g., regular intervention, routine rehabilitation training, waiting list, sham Qigong, usual care, no intervention); (iv) outcomes - cognitive ability, shoulder joint function, anxiety, depression, fatigue, sleep quality, quality of life; and (v) study design - randomized controlled trial.

**Results:**

From January 2013 to December 2023, we included a total of 16 RCTs involving 1247 patients. A total of 647 patients were in the experimental group and were treated with Tai Chi and Baduanjin, while 600 patients were in the control group and were treated with traditional methods. The results of our meta-analysis indicate that Tai Chi and Baduanjin yield outcomes that are comparable to those of traditional treatment methods. Specifically, Tai Chi and Baduanjin significantly increased cognitive function, increased shoulder joint function, improved sleep quality indicators and improved quality of life indicators. Furthermore, Tai Chi and Baduanjin significantly reduced anxiety symptoms, depression symptoms, and fatigue symptoms among breast cancer patients. Sensitivity analysis was performed, a funnel plot was constructed. No publication bias was indicated by Egger’s or Begg’s test.

**Conclusion:**

Overall, Tai Chi and Baduanjin are viable and effective nonpharmacological approaches for treating breast cancer patients, as they yield better results than traditional treatment methods. However, these findings should be interpreted with caution due to the limited number of controlled trials, small sample sizes, and low quality of the evidence.

**Systematic review registration:**

https://www.crd.york.ac.uk/PROSPERO/, identifier CRD42023469301.

## 1 Introduction

### 1.1 Description of the condition

In 2020, the estimated number of new breast cancer cases and deaths increased respectively to 2.3 million(11.6%) and 685,000(6.9%) globally, among which China ranked first in new cases and deaths worldwide, accounting for approximately 18.4% of total new cases and 17.1% of total deaths globally in 2020 (1, 2). The high incidence and mortality rate have imposed a substantial burden on healthcare systems of countries with low resources (3). When women develop breast cancer, they experience a range of related painful experiences, such as anxiety (4, 5), depression (5, 6), fatigue (7–9), cognitive impairment (10, 11), shoulder function impairment (12, 13), sleep problems (14, 15), and poor quality of life (QoL) (16, 17). Conventional treatments such as surgery for breast cancer may have negative effects on survivors of breast cancer (18). Specifically, recent research has shown that surgery may lead to sleep disorders (19), life-threatening bleeding or infections (20), flap necrosis (21) and joint damage (22) in a significant percentage of patients with breast cancer. These side effects negatively affect survivors’ overall well-being and functionality, ultimately affecting their quality of life (17). Moreover, breast cancer survivors may face various complications after conventional treatments, such as surgery (total mastectomy and breast conservation), chemotherapy, and radiotherapy (23). Furthermore, breast cancer has been reported to be a risk factor for depression (6). Due to the alarming increase in the number of breast cancer patients worldwide and the lack of effective drug treatments for these diseases. Therefore, it is highly emergent to develop non-drug therapies and apply in the field of social work for the cancer patients are highly necessary. It may slow the distress of physical and psychological in breast cancer patients to combat personal and socioeconomic problems, to address the alarming increases in the incidences of cancer patients worldwide, and to overcome the lack of effective drug treatments for such diseases.

### 1.2 Description of the Taiji and Baduanjin invention

Nowadays, people’s attentional capabilities are increasingly strained by environmental factors such as time pressure (24) or multiple task demands (25), or even professional requirements (26). Since multitasking demands preoccupy large parts of people’s daily routines, the question of how to manage or to recover from the strain imposed by overload has become increasingly important, both for researchers and practitioners (27), Especially women (28). Formally, rest breaks are defined as temporal interruptions of an activity, serving the purpose of regenerating mental functions (29, 30). Conceptually, there are three fundamental aspects that are connected to taking a break, depending on the particular context: to find distance, to change activity mode (e.g., from thinking to sensing), and to recover or regain energy levels (31).

The practice of meditation has seen a tremendous increase in the western world since the 60s (32). Scientific interest in meditation has also significantly grown in the past years (33) and increasing evidence suggests the efficacy of meditation in health care and the field of stress management (34) and some potency to enhance positive feelings (35) increase pain tolerance, and reduce anxiety (36). TaiChi and Baduanjin also belong to meditation practice (37), This are different from other physical exercises like light gymnastics (38).

Unlike in western cultures, the Chinese felt that individuals are part of a closely knit collectivity, whether a family or a village, and that the behavior of the individual should be guided by the expectations of the group (39). The relationship view versus the rule stance is well illustrated by the difference between the holistic approach to medicine characteristic of the Chinese and the effort to find effective rules and treatment principles in the West. Surgery was common in the West from a very early period because the idea that some part of the body could be malfunctioning was a natural one to the analytic mind (39). But the idea of surgery was “heretical to ancient Chinese medical tradition, which taught that good health depended on the balance and flow of natural forces throughout the body” (39).

Originating in China, Tai Chi is a mind-body physical activity known as “meditation in motion”, and it has been practiced by the Chinese for centuries (40). Tai Chi is based on Chinese martial arts and integrates meditation elements, thereby enhancing well-being and promoting physical and mental health (41). The gentle, mindful, slow, and continuous movements of Tai Chi make it a preferable complementary program for promoting wellness (42, 43). Considerable empirical evidence supports the numerous health benefits associated with regular Tai Chi practice (44–46). To address these challenges in terms of quality of life and mental health, Baduanjin exercise, known as Qigong exercise, has been recommended as an effective treatment method for patients after surgery (47–49). Baduanjin comprises eight distinct sections of movements that are performed routinely. This traditional Chinese exercise combines controlled breathing with bodily movements, aiming to improve physical fitness and mental well-being. Unlike traditional exercise methods, Baduanjin emphasizes the need for a balanced approach that not only enhances physical strength but also nurtures psychological well-being, focusing on harmonious physical and mental integration (47). Furthermore, Baduanjin exercise is easy to perform without equipment or field restrictions. Baduanjin is free of equipment or field restrictions, so it is easy for people to learn and practice (47). After the founding of the Chinese Health Qigong Association, Baduanjin underwent modifications to accommodate the needs of diverse individuals, particularly those who suffer from physical or psychological sickness (50). To date, people have developed numerous therapeutic methods. Among them, Tai Chi therapy (42, 47) and Baduanjin (47, 51, 52) therapy are nonpharmacological treatment methods that have been advocated due to their lack of side effects.

Recent meta-analyses have shown potential benefits of Taiji therapy for anxiety (53–55), fatigue (56), cognitive impairment (55), shoulder function (54), sleep problems (57, 58) and quality of life (QoL) (59). It is necessary to examine the potential benefits of these therapies. In conclusion, Tai Chi and Baduanjin therapy have been regarded as a positive treatment strategies for breast cancer patients in terms of cognitive function, shoulder joint function, and mental health. Moreover, they have also been widely used in health-related subjects due to their positive effects (59, 60).

### 1.3 Tai Chi and Baduanjin therapy on breast cancer patients

In addition to genetic factors, ageing (61), family history (62), reproductive factors (63), oestrogen (64), and lifestyle (65) are five significant risk factors for breast cancer. However, lifestyle is the only one of these risk factors that is modifiable. Tai Chi (54, 56) and Baduanjin (47, 60) have garnered increasing recognition as impactful lifestyle choices that can help prevent and improve breast cancer outcomes. It may also serve as a beneficial complement to cancer treatment, reducing the risk of both breast cancer-specific mortality and overall mortality (66, 67). The Tai Chi and Baduanjin interventions are increasingly used in oncology (48) to achieve psychosocial stabilization and provide support for cancer patients (58).

### 1.4 Research gap and aim

Firstly, there are inconsistent clinical results regarding the effects of Taijiquan and Baduanjin therapy on cognitive ability, fall prevention, shoulder joint ability and mental health. Myers et al. (68) conducted a systematic review of breast cancer patients and reported that Tai Chi improved cognitive ability. Meng et al. (69) conducted a systematic review of breast cancer patients and reported that Baduanjin improved cognitive ability. However, Wei et al. (70) find Tai Chi failed to show any between-group differences in cognitive function. Luo discovered Tai Ji had positive effects on shoulder function and strength of arm in breast cancer patients compared with the non-exercise therapy (54). Fong et al. find Tai Chi showed a favorable effect of on pain and ROM of the shoulder joint, but not on hand grip strength, flexibility, and upper limb function compared with no treatment (71). A study (70) revealed that Taijiquan and Qigong can act as primary interventions for balance training and fall prevention. However, Li reported that there was no significant decrease in falls for strength training or Tai Chi Quan compared to the stretching control group among postmenopausal women receiving chemotherapy (58). Luo et al. reported that Tai Chi therapy was effective for improving mental health in breast cancer patients (72). Ye et al. (73) reported that Baduanjin therapy was effective for improving mental health in breast cancer patients. As reported by several researchers (47, 74, 75), Tai Chi and Baduanjin can significantly improve mental health. However, another observational study detected no significant time × group interaction effects on stress and mental health (76). Therefore, recent meta-analyses have reported conflicting results. Luo et al. (54) discovered that Tai Chi can decrease the fatigue symptom to breast cancer patients while Liu et al. (56) Hold the opposite view. Sameh Gomaa et al. find Tai Chi improved sleep quality and depressive symptoms (77). Wei et al. discovered Tai Chi failed to show any between-group differences in sleep quality and depression symptoms (70). Liu et al. demonstrated that tai chi is no different from conventional supportive care interventions in improving fatigue, sleeping quality, depression symptom at either 3 months or 6 months (54). Clinical data examining the effects of Tai Chi and Baduanjin on cognitive impairment, shoulder function impairment, Prevent falls, mental health fatigue, sleeping quality and depression symptom remain controversial. A more comprehensive review is needed to systematically evaluate the effects of Tai Chi and Baduanjin on the physical and mental health of breast cancer patients.

Secondly, many studies included indicated that Tai Chi and Baduanjin had positive effects on cognitive function, shoulder joint function, depression, anxiety, fatigue, sleep quality, and quality of life, but none included the effect of both Tai Chi and Baduanjin therapy on seven outcome measures. Tai Chi, breast cancer patients, cognitive function, shoulder joint function (54) depression symptom (78), anxiety symptom (79), fatigue symptom (78), sleep quality (80), and quality of life (81). Wei observed Baduanjin exercise has a certain preventive effect on the decline of subjective cognitive ability of breast cancer patients during chemotherapy clearly (70). Baduanjin, breast cancer patients, cognitive function (82), shoulder joint function (58), depression symptom (83), anxiety symptom (83), fatigue symptom (84), sleep quality (70), and quality of life (60). This study investigates the effects of Tai Chi and Baduanjin on the cognitive ability, shoulder joint ability and mental health of breast cancer patients simultaneously.

Thirdly, it is important to investigate not only the effects of Tai Chi and Baduanjin but also the variables that may influence their effectiveness, including duration of treatment and target population. In this research, we used weeks and continental plate to divide Sub-group analysis, which was not available in previous studies. To address this gap, we conducted a comprehensive systematic review and meta-analysis to evaluate the impact of Tai Chi and Baduanjin on training breast cancer patients.

## 2 Materials and methods

This review was conducted in accordance with the Preferred Reporting Items for Systematic Reviews and Meta-Analyses (PRISMA, 2020) guidelines, as detailed in the **Multimedia Appendix 3** Abstracts checklist and **Multimedia Appendix 4** Checklist 2020 (85). The study was also registered in PROSPERO (CRD42023469301).

### 2.1 Selection criteria

For this study, eligibility criteria were established based on the PICOS principles. (1) P: The subjects had to exhibit at least one indicator of cognitive function, shoulder joint function, or mental health, and they had to be 18 years of age or older. (2) I: Tai Chi and Baduanjin were implemented among breast cancer patients in the experimental group without restrictions on the time of intervention. (3) C: The control group received a different intervention (e.g., regular intervention, routine rehabilitation training, waiting list, sham qigong, usual care, no intervention). (4) O: The outcomes included cognitive function, shoulder joint function and mental health in breast cancer patients. (5) S: The type of study was RCTs. The exclusion criteria were as follows: (1) reviews, case reports, non-RCTs, or articles without full-text availability; (2) animal experiments or duplicate publications; and (3) unavailable or incomplete data. Furthermore, we used the PICOS principles to identify eligible studies (**Multimedia Appendix 1**). All studies included were published in Chinese or English, and studies that interpreted results from the perspective of breast cancer patients were considered eligible.

### 2.2 Search strategy

To identify relevant literature, the following databases were searched: the UWE Library database, PubMed, MEDLINE, Embase, the Cochrane Library, Scopus, PsycINFO, SinoMed, Wanfang Data, China National Knowledge Infrastructure (CNKI), Yiigle, Wanfang MED ONLINE, and Web of Science. The search terms varied slightly across databases. Keywords such as “Cognitive Function or Shoulder Joint Function or Mental Health or Depression or Anxiety or Fatigue or Sleep Quality or Quality of life”, and “a pilot study or Randomized Controlled Trial or RCT,” and “Tai Chi and Baduanjin,” as well as “Breast Cancer Patients or Breast Cancer Women” were used to retrieve articles published from January 1, 2013, to December 1, 2023. The “snowball” method was employed to trace the references of the included studies. Moreover, the references of the included studies were manually searched to identify additional eligible articles. Academic unpublished literature was considered ineligible. The retrieval strategy for the PubMed database is provided in **Multimedia Appendix 2**.

**Figure 1:**
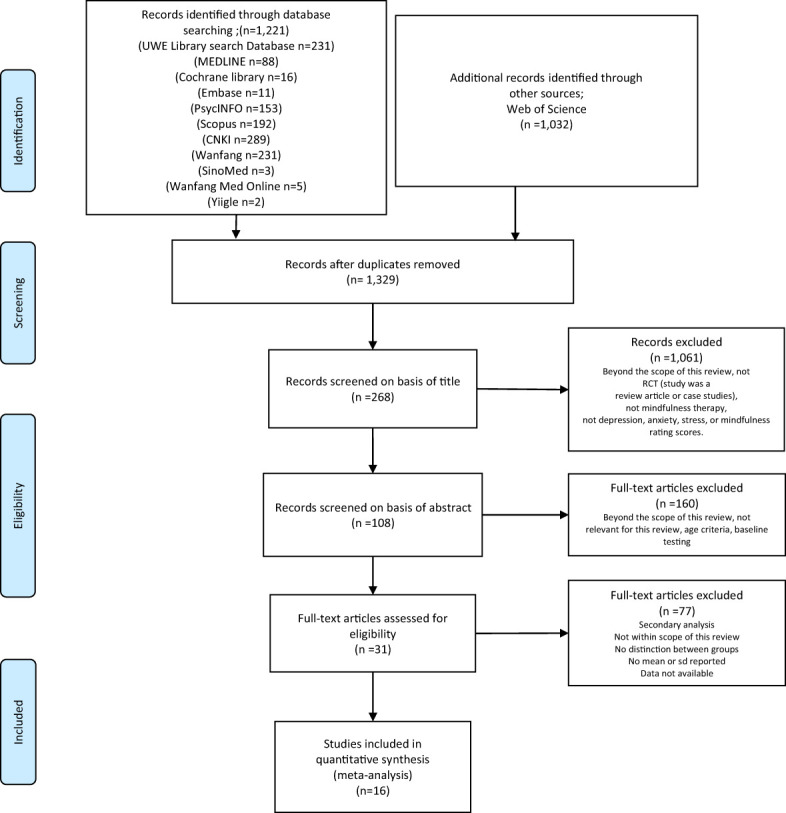
Study selection flowchart (PRISMA, 2020).

The search strategies are presented in **Figure 1**. Four researchers (Zuo Xinyi, Tang Yong, Chen Yifang and Zhou Zhimiao) screened all the literature for eligibility. After the removal of duplicates, the initial screening of all studies was based on abstracts and titles. Subsequently, the researchers read the full texts of the remaining articles according to the predefined inclusion and exclusion criteria. Finally, they extracted data from the selected literature. Additionally, we retrieved grey literature (opengrey.eu) to further identify related publications.

### 2.3 Data extraction

After removing duplicate studies (using EndNote X9), four reviewers (Zuo Xinyi, Tang Yong, Chen Yifang and Zhou Zhimiao) independently screened the abstracts and titles of the studies. Subsequently, the full texts of the remaining studies were evaluated according to the predefined inclusion and exclusion criteria.

The first authors (Zuo Xinyi and Chen Yifang) used a modified version of the data extraction form in the Cochrane Handbook for Systematic Reviews of Interventions (86) to extract study characteristics. They also extracted data on the content, design, and delivery features of the interventions (definitions provided in the “Introduction”) by a modified template version for intervention description as well as replication (TIDieR) checklist and guide (87). On a random sample of three studies, the forms were first tested and then used by the first authors (Zuo Xinyi and Chen Yifang) for the completion of data extraction. Once the study was completed, the accuracy of the process was verified by the second author (Zhou Zhimiao). Finally, through thematic analysis (88), we determined in advance the content of the author, year, country, publication type, study design, sample size, mean age (SD), age, MMSE score, health condition, population group, setting, results as reported in the studies, and conclusions (**Table 1**); author, year, intervention type, modes of delivery, materials, procedures, activities, and/or processes, format of the intervention delivery, location, duration of intervention, length of sessions, frequency of sessions, intensity, measurement, follow-up, and Jadad score (**Table 2**).

**Table 1:** Study characteristics and findings of the included studies.

Author(year)	Country	Publication type	Study design	Sample size			Age/Mean age (SD)		Health condition	Population group	Setting	Conclusion
				Total	IG^1^	CG^2^	IG	CG				The interactive game can improve senile dementia patients` cognitive function and daily living ability.
1. Han et al., 2017 (89)	China Shanxi	JA^3^	RCT^4^	60	30	30	39-55(47.83)	37-55(46.23)	BC^5^	Chemotherapy patients one month after breast cancer surgery	clinical	Baduanjin training can significantly improve the anxiety and of mental health status patients after breast cancer surgery.
2. Li et al.2017 (90)	China Shanxi	JA	RCT	61	31	30	37-57(47.31)	34-56(45.43)	BC	Diagnosed breast cancer who requiring radiotherapy after chemotherapy	clinical	Baduanjin intervention can improve the anxiety and depression & improve the quality of life of patients
3 Li et al., 2013 (91)	China Fujian	JA	RCT	N =57	29	28	47(47.56)	47(47.56)	BC	Pathology confirmed breast cancer, and modified radical operation	clinical	Tai Chi Yunshou training has a certain effect on improving the quality of life of patients.
4 Lv et al., 2015 (92)	China Henan	JA	RCT	N=149	100	49	32-65(48.61)	32-65(48.61)	BC	Pathologic diagnosis and voluntary modified radical treatment of unilateral breast cancer	clinical	Tai Chi have potential advantages in promoting the postoperative rehabilitation of breast cancer patients and improving the quality of life of patients after surgery.
5 Wang Hongying, 2015 (93)	China Wuhan	JA	RCT	N =149	75	74	43-59(51.4)	42-58(50.58)	BC	Clinical pathology examination	clinical	The exercise mode of Tai Chi and rehabilitation exercises has a positive effect on improving the mental health of breast cancer patients after surgery.
6 Wang et al., 2016 (94)	China Wuhan	JA	RCT	N=96	48	48	46-60(53.64)	44-59(51.74)	BC	Confirmed breast cancer on pathological examination, with modified radical resection	clinical	
7 Yang et al., 2022 (95)	China Henan	JA	RCT	N=88	44	44	37-78(58.24)	36-78(57.86)	BC	Gave surgical resection and postoperative chemotherapy intervention	clinical	Baduanjin exercise can improve post-surgery treatment compliance, limb function, health status, quality of life, mood, nursing satisfaction, and have minimal side effects for breast cancer patients.
8 Liao et al., 2022 (82)	China Guangzhou	JA	RCT	N=68	33	35	46-60(53.12)	46-63(54.63)	BC	Diagnosis with stage I–III breast cancer for 6 months to 8 years prior to recruitment	clinical	Baduanjin exercise improved the quality of life and sleep of breast cancer patients.
9 Larkey et al., 2015 (96)	The United State	JA	RCT	N =87	42	45	48-66(57.7)	50-68(59.8)	BC	Diagnosed with Stage 0-III breast cancer	clinical	Both Qigong and Tai Chi can improved depression and sleep dysfunction among breast cancer survivors.
10 Larkey et al., 2016 (97)	The United State	JA	RCT	N =87	42	45	48-66(57.7)	50-68(59.8)	BC	Diagnosed with Stage 0-III breast cancer	clinical	Both Qigong and Tai Chi could enhance the quality of life, cognitive function, and physical activity patterns in women with a history of breast cancer.
11 Thongteratham et al., 2015 (98)	Thailand	JA	RCT	N=30	15	15	54-65(60.3)	55-65()61	BC	First diagnosis with stage 0-IIIb breast cancer	clinical	The study suggests that Tai Chi Qi Qong benefits Thai women with breast cancer.
12 Huang et al., 2016 (99)	China Taiwan	JA	RCT	N=62	31	31	18-65(51.4)	18-65(51.4)	BC	Adult women with breast cancer who were about to start chemotherapy	clinical	The study preliminarily suggests SQG and NSQG may improve frailty and QOL in breast cancer patients undergoing chemotherapy.
13. Fong et al., 2013 (100)	China HongKong	JA	RCT	N=23	11	12	48-68(58.3)	49-58(53.8)	BC	Having received a mastectomy with or without adjutant chemotherapy or radiotherapy	clinical	Tai Chi and Qigong training might improve shoulder muscular strength and functional wellbeing in breast cancer survivors.
14 Loh et al., 2014 (101)	Malaysia	JA	RCT	N =64	32	32	18-65(41.5)	18-65(41.5)	BC	Had a primary diagnosis of early stage (I-II) breast cancer	clinical	Qigong mildly enhanced life quality in cancer survivors.
15 Wei et al., 2022 (70)	China Shanghai	JA	RCT	N =70	35	35	43-60(52)	50-62(55)	BC	Female patients newly diagnosed with stage I to III BC and scheduled to receive chemotherapy	clinical	This pilot study showed that Baduanjin exercise improves cognition and quality of life in Chinese breast cancer patients undergoing chemotherapy.
16 Chen et al., 2013 (102)	China Shanghai	JA	RCT	N=96	49	47	29-58(45.3)	25-62(44.7)	BC	Women with stage 0–III breast cancer who were had undergone breast surgery	clinical	Qigong help to improve quality of life and reduce depressive symptoms in women undergoing radiotherapy for breast cancer.

1 IG, Intervention Group.

2 CG, control group.

3 JA, Journal article.

4 RCT, Randomized controlled trial.

5 BC, breast cancer.

**Table 2:** Intervention characteristics—Adapted from the template for intervention description and replication (TIDieR) checklist and guide.

Author, year	Intervention type		Content (what)—the materials, procedures, activities, and/or processes	Delivery (who, where, when, how much)—format of the intervention delivery, the location, duration of intervention, length of sessions, frequency of sessions, intensity	Measurement	Follow-up	Jadad score
	IG^a^	CG^b^					
1. Han et al., 2017 (89)	BaDuanJin training	regular intervention	Under the guidance of the head coach, 5 students became auxiliary coaches responsible for guiding patients to perform Eight Section Brocade training in the hospital square until they mastered the correct movements. The discharged patient trains for 20 minutes every afternoon, 5 times a week and daily follow-up.	Format: group Location: public hospital Duration: 3 month Length: 20 min Frequency: 5 times/week Intensity: move follow with the DVD of baduanjin training	Anxiety: SAS^c^	Baseline, Post-intervention	4
2 Li et al., 2017 (90)	BaDuanJin training	regular intervention	A nurse became the main trainer and led 5 interns jointly conducted a one week Eight Section Brocade training for 31 patients, including group exercises at the hospital square, exercises on CDs published by the Fitness Qigong Management Center of the General Administration of Sport of China, and follow-up on the completion status of patients through phone calls.	Format: group Location: public hospital or Home Duration: 3 month Length: no record Frequency: 5 times/week Intensity: move follow with the DVD of baduanjin training	Anxiety:SAS Depresstion: SDS^d^	Baseline, Post-intervention	5
3 Li et al., 2013 (91)	TaiChi cloudyhand	regular intervention	Starting 1-2 weeks after surgery, shoulder joint activities will be carried out, with the arm swinging back and forth centered around the shoulder. Daily activities will gradually be performed. Train 1-3 times a day for 20-30 minutes each time. The experimental group began Tai Chi training 7 days after surgery. Three nurses provide Tai Chi exercise guidance to patients through “Tai Chi Exercise”, helping them master the correct posture of Tai Chi cloud hand movements, and lead patients to 30 minutes of training every day in the hospital’s aerial garden corridor.	Format:group Location: public hospital Duration: 6 month Length: 30min Frequency: 14 times/week Intensity: move follow with nurse order	Quality of life: WHOQOLBREF^e^ Shoulder joint function: Lovett	day 7, 1 month, 3 month, 6 month.	5
4 Lv et al., 2015 (92)	TaiChi & Baduanjin	regular intervention	A week of basic training, after the patient can accurately master the movements and breathing essentials into the exercise intervention period. Practice for 60min and 3 times a week for 6 months.	Format:group Location:community Duration: 3 month Length: 60 min Frequency: 3 times/week Intensity: learn from order and do it by themselves	Quality of life: SF-36^f^ Shoulder joint funtion: Constant-murley	day 10, 1 month, 3 month.	4
5 Wang Hongying 2015 (93)	TaiChi	Routine rehabilitation training	Transitional training at least twice a day for 20-30 minutes each time. After 10 days, the intervention group patients can undergo Chen’s Tai Chi exercise, once in the morning and once in the evening, for 20 minutes each time. After discharge, the patients still need to follow the exercise until 6 months after surgery. Conduct two phone follow-up or home visits per week to continue routine rehabilitation training and Tai Chi exercise.	Format: group Location: public hospital or Home Duration: 6 month Length: 20min Frequency: 14 times/week Intensity: On-site teaching and broadcast Taijiquan video, the chief nurse on time patrol	Anxiety: SAS	day 10, 1 month, 3 month, 6 month.	4
6 Wang et al., 2016 (94)	Tachi & regular intervention	regular intervention	Tai Chi exercises are jointly developed by Tai Chi trainers and head nurses, including on-site teaching and playing Tai Chi videos. The patient needs to undergo two routine rehabilitation exercises and two Tai Chi exercises per day, each lasting 20 minutes. After discharge, the nurse will conduct telephone follow-up or home visits twice a week to ensure that the patient continues to receive routine rehabilitation training and Tai Chi exercises.	Format: group Location: public hospital or Home Duration: 3 month Length: 20min Frequency: 14 times/week Intensity: learn from order and do it by themselves	Shoulder joint funtion: Neer Quality of life: FACT-B^g^	day 10, 1 month, 3 month, 6 month.	4
7 Yang et al., 2022 (95)	Traditional Chinese medicine rehabilitation physiotherapy&Baduanjin	regular intervention	A fitness qigong called Ba Duan Jin have 10 movements that can help patients relax and maintain natural breathing. Before discharge, the therapist will guide the patient and their family to master relevant physical therapy and exercise methods, and develop a training plan to follow up on the patient’s training situation.	Format: group Location: public hospital or Home Duration: 3 month Length: no record Frequency: 7 times/week Intensity: learn from video guided by professional	Shoulder joint funtion: Constant-murley Quality of life: FACT-B	Baseline, Post-intervention	4
8 Liao et al., 2022 (82)	Baduanjin	usual care	The Baduanjin program was composed of 90 min per session with 2 sessions per week (Monday and Wednesday) for 12 weeks.	Format: group Location: public hospital or Home Duration: 3 month Length: 90 min Frequency: 2 times/week Intensity: learn from 2 experts	Sleep quality: PSQI Quality of life: EORTC QLQ-c30^h^	Baseline, Post-intervention	4
9 Larkey et al., 2015 (96)	TaiChi/Qigong	sham Qigong	This research project has two courses led by a nurse and a professor of exercise physiology. There are two meetings per week in the first two weeks, and only one meeting per week thereafter. Participants are required to practice at home for 30 minutes per week for a total of 5 days, and the frequency, number of minutes, and level of effort of the practice must be recorded.	Format: group Location: Home Duration: 3 month Length: 30 min Frequency: 5 times/week Intensity: learn from 2 experts	Fatigue: FSI Sleep quality: PSQI Depression: BDI^i^	Baseline, Post-Intervention, 3 Month Follow up	5
10 Larkey et al., 2016 (97)	TaiChi/Qigong	sham Qigong	Professionals experienced in leading exercise sessions with cancer patients taught both Qingong and Tai Chi Easy56 practices (TCE) exercises interventions. Sessions were sixty minutes long, delivered over 12 weeks, meeting twice a week for the first two weeks to provide the opportunity to learn the practices well, then once a week for the remainder of the period. Participants were asked to practice at home at least 30 minutes a day, 5 days per week and to keep a log of the frequency, minutes of practice and level of exertion.	Format: group Location: Home Duration: 3 month Length: 30 min Frequency: 5 times/week Intensity: learn from 2 experts	Cognitive function: FACT-cogPCI^j^ Quality of life: SF36	Baseline, immediately after the 12-week intervention and again at 12 weeks post-intervention.	6
11 Thongteratham et al., 2015 (98)	TaiChi/Qigong	usual care	Each 60-minute TCQQ session is divided into three phases: 1) warm-up (extend muscle for 5 minutes), 2) exercise (18-form TCQQ practicing for 45-50 minutes), and cool-down(decreasing exercise to normal for 5-10 minutes). The 18-form TCQQ was grouped into 3 sets (6-form/set) aiming to assist participants’ memory. The participants practiced the 18-forms at the 4th week of the program. A weekly follow-up telephone call was done by the PI to monitor.	Format: group Location: Home Duration: 12 week Length: 60 min Frequency: 5 times/week Intensity: learn from TCQQ.The TCQQ Program was developed by the researchers, based on TC literature and research and was validated by 3 experts, a psychologist, a physical therapy instructor, and an advanced practice nurse (APN) in medical-surgical nursing	Quality of life: FACT-B Fatigue: FSI^k^	Baseline, 6week, 12 week	6
12 Huang et al., 2016 (99)	Sporting Qigong	usual care	This study invited two qigong experts, two general surgeons, and two oncology case managers to help patients exercise. Participants were asked to exercise three times a week for 12 weeks, each lasting 30 minutes. The researchers also contacted all participants by phone to encourage them to exercise regularly.The patients were asked to complete finger and palm exercises, shoulder exer_x0002_cises, hand lifting, hair combing, pendulum exercises, wall hand climbing, pulley tugging, chest flies, towel exercises, rod lifting, pushing on the wall, and putting on a brassiere.	Format: group Location: hospital & home Duration: 12 week Length: 30 min Frequency: 3 times/week Intensity: All participants have received at least one training session before starting their exercise or qigong program. The number of training courses is based on the patient’s ability to fully intervene. They are required to undergo postoperative exercise	Quality of life: Physical and mental components for QOL	baseline, 1 month, 3 months	4
13. Fong et al., 2013 (100)	Tai Chi Internal Qigong	regular intervention	This exercise will be conducted 3 times a week for 1 hour each time. It includes 18 movements aimed at helping the body relax, reduce stress, and enhance breathing awareness. These movements include center of gravity transfer, arm swing, boxing, and mild stretching of various parts of the body, which require breathing control under professional guidance.	Format: group Location: the Sports Training and Rehabilitation Labo_x0002_ratory Duration: 6 month Length: 60 min Frequency: 3 times/week Intensity: trained by experts	Cognitive fouction: FACT-B Shoulder joint function: Shoulder active Rom	Baseline, Post-Intervention	4
14 Loh et al., 2014 (101)	internal Qigong (Zhi Neng Qigong) programme	usual care	Invite participants to participate in an 8-week qigong course each week, taught in person by qualified qigong masters. Warm up 10 minutes before each class, followed by 70 minutes of main exercise, and remain calm for 10 minutes at the end of the class. The course includes two 5-minute breaks. This course teaches the basic steps of empowering qigong, including three different starting sequence postures. Attendees need to attend five meetings to master their posture and pace.	Format: group Location: community Duration: 8 week Length: 60 min Frequency: 1 times/week Intensity: trained by experts face to face	Cognitive function:FACT-B	Baseline, Post-Intervention, Follow-up study (12 month after baseline)	5
15 Wei et al., 2022 (70)	BaDuanJin training	usual care	This is a qigong training consisting of 10 postures, with professional trainers providing training guidance and video demonstrations. After the first hospital meeting before starting chemotherapy, it is recommended that patients undergo video exercise at home, 5 times a week for half an hour each time, for a period of 12 weeks. The training includes stretching joints, inhaling and exhaling, 2 minutes of muscle relaxation, and two 12 minute Eight Section Brocade exercises.	Format: group Location: hospital & home Duration: 12 week Length: 30 min Frequency: 5 times/week Intensity: trained by experts and video tape	Quality of life: FACT-B Anxiety: HADS^l^ Depression: HADS	pre-intervention, 4 weeks, 8 weeks, and 12 weeks	3
16 Chen et al., 2013 (102)	qigong sessions	no intervention	Women in the qigong group attend 5 sessions 40 minute qigong classes per week for 5-6 weeks. These courses are taught by a government licensed master of traditional Chinese medicine and qigong. The number of participants may vary, ranging from 1 to 10. Participants will receive a DVD containing program records of qigong and some printed materials, encouraging them to complete the course without a qigong master.	Format: group Location: hospital & home Duration: 3 month Length: 40 min Frequency: 1 times/week Intensity: trained by experts and video tape	Cognitive function: FACT-G^m^ Depression: CES-D^n^ Sleep quality: PSQI^o^	baseline, middle, Last week of treatment, 1 months later, and 3 months later.	3

a IG, Intervention Group.

b CG, control group.

c SAS, self-rating anxiety scale;

d SDS, self-rating depressive scale;

e WHOQOLBREF, Brief table of quality of life assessment recommendation by WHO;

f SF-36, The 36-item Medical Outcomes Survey Short Form;

g FACT-B, functional assessment of cancer therapy—breast;

h EORTC QLQ-C30, European Organization for Research and Treatment of Cancer Quality of Life Questionnaire Core 30;

i BDI, the 20-item Beck Depression Inventory;

j Fact-Cog PCI, the Functional Assessment of Cancer Therapy-Cognitive Function perceived cognitive impairment;

k FSI, The Fatigue Symptom Inventory;

l HADS, hospital anxiety and depression scale;

m FACT-G, The Functional Assessment of Cancer Therapy–General;

n CES-D, the Center for Epidemiologic Studies-Depression;

o PSQI, The Pittsburgh Sleep Quality Index.

The following data were extracted from the included studies: (1) the information of the literature included (name of author, publication year, country, publication type and study design); (2) the characteristics of the subjects (sample size, participant mean age, participant age, Mini-mental State Examination (MMSE) score, participant health conditions and population group); (3) the interventions (setting, intervention type, design (how)—the modes of delivery, content (what)—the materials, procedures, activities, and/or processes, delivery (who, where when, how much)—format of the intervention delivery, the location, duration of intervention, length of sessions, frequency of sessions, intensity); (4) the information on quality of the study (Jadad score); and (5) the main outcomes (results as reported in studies, conclusion, measurement and follow-up).

### 2.4 Quality and bias assessment

Using the Cochrane Collaboration Risk of Bias tool, authored by Higgins et al. (86) in 2008, four researchers—Zuo Xinyi, Tang Yong, Chen Yifang and Zhou Zhimiao—independently evaluated the potential for bias in the included studies. Furthermore, to assess the overall quality of the literature, we employed the Jadad scale (103). This risk of bias tool encompasses seven distinct domains: 1. allocation concealment, 2. random sequence generation, 3. blinding of subjects and experimenters, 4. blinding of outcome assessors, 5. selective reporting, 6. integration of resulting data, and 7. other potential sources of bias. Each study was classified as having an unclear, low, or high risk of bias for each domain. Additionally, to assess the degree of publication bias, we conducted the Egger and Begg tests; through these comprehensive assessments, we aimed to ensure the reliability and validity of our findings (76).

### 2.5 Statistical analysis

Data analysis was conducted utilizing Review Manager 5.3 software and Stata 15.1. To visually represent the findings, forest plots were generated. The studies included in the analysis measured outcomes as continuous variables, with the same indicator assessed using diverse tools. To standardize these measurements, the outcomes are expressed as standard mean differences (SMDs). Statistical significance was determined at α = 0.05. Through this rigorous analytical process, we aimed to provide a comprehensive understanding of the results. Heterogeneity was evaluated using I² statistics, with categories defined as high (>75%), moderate (50-75%), low (<50%), or as outlined (104) in 2002. When high heterogeneity was observed, a sensitivity analysis was conducted using the leave-one-out approach to pinpoint potential sources. During this analysis, multiple weeks were compared as subpoints to ensure the robustness of the findings. Additionally, Begg’s test (105) and Egger’s test (106) in 1997 were utilized to assess the likelihood of publication bias, thereby enhancing the overall reliability of our results. When the meta-analysis included at least 10 studies, a funnel plot (107, 108) was used to assess potential publication bias. Specifically, this study focused on examining the standard mean difference (SMD) along with its corresponding 95% confidence interval (CI) (109). If the overall effect yielded a p value less than 0.05, it was interpreted as statistically significant evidence favouring the effects of MBIs.

### 2.6 Subgroup analyses

We conducted subgroup analyses on the basis of intervention country (inside or outside China) and intervention duration (weeks).

## 3 Results

### 3.1 Selection of studies

**Figure 1** outlines the study selection process, and the results are presented in **Figure 1**. After searching through thirteen databases, a total of 2,253 records were found. All studies were imported into EndNote X8 (Bld, 10063) (110), and duplicates were removed. Following the removal of 924 duplicates and a rigorous screening process that eliminated 1,313 articles, 16 trials (70, 82, 89–102) with 1,256 participants were ultimately included. Studies were excluded if they did not report sd values (75), were missing data for the control group (111), were review articles (68), or were not within the scope of this meta-analysis (112). All the studies included indicated that Tai Chi and Baduanjin had positive effects on cognitive function, shoulder joint function, depression, anxiety, fatigue, sleep quality, and quality of life. The primary outcomes of interest were as follows: cognitive function scores, as assessed by the Functional Assessment of Cancer Therapy-Cognitive Function Perceived Cognitive Impairment (Fact-Cog PCI), The Functional Assessment of Cancer Therapy-Breast (FACT-B), The Functional Assessment of Cancer Therapy-General (FACT-G); shoulder joint function, as assessed by the shoulder active Rom, Lovett Constant-Murley, Neer; depression symptoms, as assessed by the Center for Epidemiologic Studies-Depression (CES-D), the 20-item, Beck Depression Inventory (BDI), self-rating depressive scale (SDS), hospital anxiety and depression scale (HADS); anxiety symptoms, as assessed by the Self-rating Anxiety Scale (SAS), Hospital Anxiety and Depression Scale (HADS); fatigue symptoms, as assessed by the British Standard Institution (BSI), The Fatigue Symptom Inventory (FSI), Multidimensional Fatigue Symptom Scale type: Primary indicator (MFSI-SF); sleep quality, as assessed by the Pittsburgh Sleep Quality Index (PSQI); quality of life, as assessed by the 36-item Medical Outcomes Survey Short Form (SF-36), European Organization for Research and Treatment of Cancer Quality of Life Questionnaire Core 30 (EORTC QLQ-C30), Brief table of quality of life assessment recommendation by WHO (WHOQOLBREF), Functional Assessment of Cancer Therapy—Breast(FACT-B), physical components of quality of life and mental components of quality of life.

### 3.2 Study characteristics

**Tables 1**, **2** present the general features of the included studies. All 16 studies were published before 2024. The sample sizes ranged from 11 to 100, and a total of 1247 breast cancer patients over the age of 18 were enrolled, with 654 participants in the experimental group and 602 in the control group. None of the breast cancer patients had been diagnosed with a psychiatric disorder. The interventions were based on Tai Chi and Baduanjin, with durations ranging from 5 weeks to 24 weeks. The weekly intervention time varied between 20 minutes and 90 minutes. Both individual and group training methods were utilized (70, 82, 89–102). All studies (70, 82, 89–102) were further categorized into Tai Chi and Baduanjin therapy.

### 3.3 Risk of bias and quality assessment

**Figure 2:**
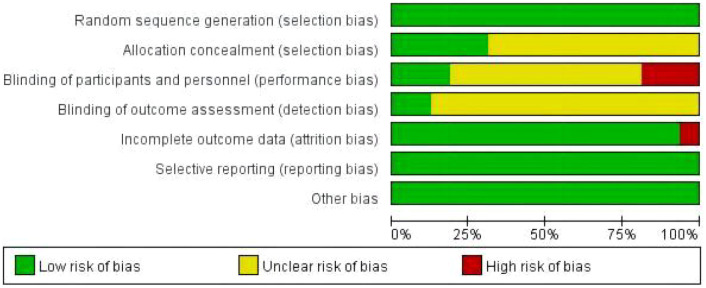
Risk of bias graph.

**Figure 3:**
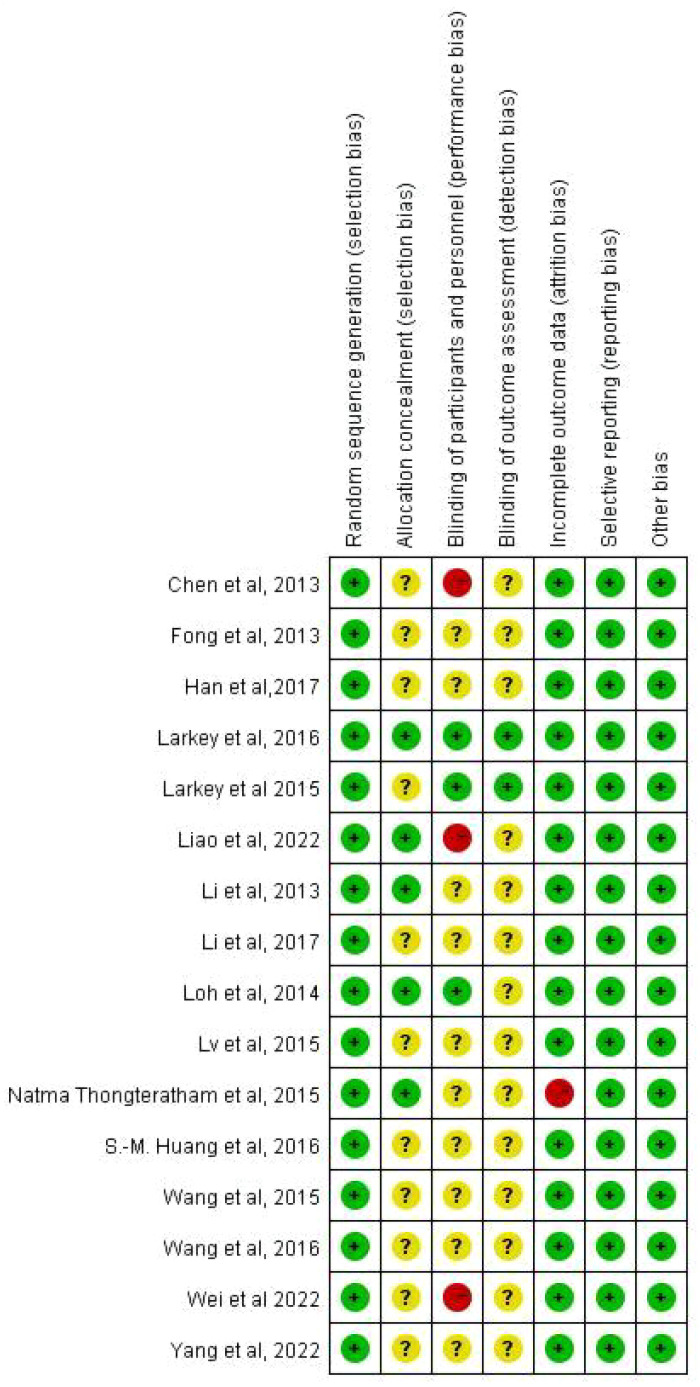
Risk of bias summary of included studies.

**Figures 2** and **3** provides an assessment of the risk of bias. All 16 articles (70, 82, 89–102) provided detailed descriptions of the randomization methods. Four studies reported the blinding method, and all of them were single-blinded trials (82, 89, 91, 95). The dropout rate was reported in two articles (89, 96). The average Jadad score for all included studies was 4.1875, indicating fair to mild quality. 

### 3.4 Meta-analyses

#### 3.4.1 Cognitive function scores

**Figure 4:**
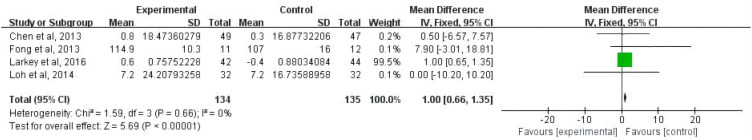
Forest plot for MMSE scores.

Four studies involving 209 breast cancer patients (141 in the experimental group and 138 in the control group) evaluated the effect of Tai Chi and Baduanjin on cognitive function in breast cancer patients. Due to the utilization of various evaluation tools, the SMD was utilized as the pooled measure for effect size. The pooled results revealed no heterogeneity among the studies (P =.66, I^2^ = 0%). As shown in **Figure 4**, the Tai Chi and Baduanjin groups had significantly higher cognitive ability scores than the control group (SMD 1.00, 95% CI 0.66 to 1.35; P<.00001).

#### 3.4.2 Shoulder joint function scores

Five studies involving 456 breast cancer patients (228 in the experimental group and 228 in the control group) evaluated the effect of Tai Chi and Baduanjin on the Shoulder Joint Function scores of breast cancer patients based on the shoulder active Rom, Lovett Constant-Murley, Neer. Due to the utilization of various evaluation tools, the SMD was utilized as the pooled measure for effect size. The pooled results revealed a high degree of heterogeneity among the studies (P=.07, I^2^ = 51%), necessitating the use of a random effects model for the meta-analysis. As shown in **Supplementary Figure 5**, there were significantly higher shoulder joint function scores in the Tai Chi and Baduanjin groups than in the control group (SMD 7.34, 95% CI 6.32 to 8.35; P<.00001).

#### 3.4.3 Anxiety indicators

Four studies involving 340 breast cancer patients (171 in the experimental group and 169 in the control group) evaluated the effect of Tai Chi and Baduanjin on anxiety symptoms among breast cancer patients based on the SAS and HADS. Due to the utilization of various evaluation tools, the SMD was utilized as the pooled measure for effect size. The pooled results revealed a high degree of heterogeneity among the studies (P<.00001, I^2^ = 90%), necessitating the use of a random effects model for the meta-analysis. As shown in **Supplementary Figure 6** the Tai Chi and Baduanjin groups had significantly lower anxiety scores than the control group (SMD - 2.22, 95% CI - 3.15 to - 1.29; P<.00001).

#### 3.4.4 Depression indicators

Four studies involving 318 breast cancer patients (158 in the experimental group and 160 in the control group) evaluated the effect of Tai Chi and Baduanjin on depression symptoms in breast cancer patients based on the CES-D, BDI, SDS, and HADS. Due to the utilization of various evaluation tools, the SMD was utilized as the pooled measure for effect size. The pooled results revealed a high degree of heterogeneity among the studies (P=.02, I^2^ = 88%), necessitating the use of a random effects model for the meta-analysis. A shown in **Supplementary Figure 7**, the Tai Chi and Baduanjin groups had significantly lower depressions scores than the control group (SMD - 1.44, 95% CI -2.46 to -0.41; P<.006).

#### 3.4.5 Fatigue indicators

Four studies involving 282 breast cancer patients (141 in the experimental group and 141 in the control group) evaluated the effect of Tai Chi and Baduanjin on fatigue symptoms in breast cancer patients based on the BSI, FSI, and MFSI-SF. Due to the utilization of various evaluation tools, the SMD was utilized as the pooled measure for effect size. The pooled results revealed a high degree of heterogeneity among the studies (P=.17, I^2^ = 41%), necessitating the use of a random effects model for the meta-analysis. As shown in **Supplementary Figure 8**, the Tai Chi and Baduanjin groups had lower fatigue scores than the control group (SMD - 1.02, 95% CI -1.52 to -0.53; P<.0001).

#### 3.4.6 Sleep quality indicators

Three studies involving 222 breast cancer patients (110 in the experimental group and 112 in the control group) evaluated the effect of Tai Chi and Baduanjin on sleep quality indicators in breast cancer patients based on the PSQI. Due to the utilization of various evaluation tools, the SMD was utilized as the pooled measure for effect size. The pooled results revealed a high degree of heterogeneity among the studies (P=.001, I^2^ = 85%), necessitating the use of a random effects model for the meta-analysis. As shown in **Supplementary Figure 9**, the Tai Chi and Baduanjin groups had higher sleep quality scores than the control group (SMD - 1.44, 95% CI -2.57 to -0.31; P=.01).

#### 3.4.7 Quality of life indicators

Nine studies involving 749 breast cancer patients (372 in the experimental group and 377 in the control group) evaluated the effect of Tai Chi and Baduanjin on breast cancer patients’ quality of life indicators based on the SF-36, EORTC QLQ-C30, WHOQOLBREF, FACT-B, physical components of quality of life and mental components of quality of life. Due to the utilization of various evaluation tools, the SMD was utilized as the pooled measure for effect size. The pooled results revealed a high degree of heterogeneity among the studies (P=<.0001, I^2^ = 93%), necessitating the use of a random effects model for the meta-analysis. **Supplementary Figure 10** shows that there was a significant increase in the quality-of-life scores of the Tai Chi and Baduanjin groups than the control group (SMD 6.94, 95% CI 5.60 to 8.27; P<.00001).

In conclusion, the included studies used the following assessment tools: the Fact-Cog PCI, FACT-B, FACT-G, shoulder active Rom, Lovett Constant-Murley, Neer, CES-D, BDI, SDS, HADS, SAS, HADS, BSI, FSI, MFSI-SF, PSQI, SF-36, EORTC QLQ-C30, WHOQOLBREF, FACT-B, physical components of quality of life and mental components of quality of life. Seven outcomes were evaluated: cognitive function, shoulder joint function, depression, anxiety, fatigue, sleep quality, and quality of life. The Tai Chi and Baduanjin groups had significantly greater total scores for cognitive function than the control group (CG) (SMD 1.00, 95% CI: [0.66, 1.35], P<.00001, I^2^ = 0%) (**Figure 4**). Compared with the control group (CG), the Tai Chi and Baduanjin groups had significantly greater total shoulder joint function scores (SMD 7.34, 95% CI: [6.32, 8.35], P<.00001, I^2^ = 51%) (**Supplementary Figure 1**). In addition, the Tai Chi and Baduanjin groups had lower anxiety scores than the CG (SMD - 2.22, 95% CI: [- 3.15, - 1.29], P <.00001, I^2^ = 90%) (**Supplementary Figure 2**). The Tai Chi and Baduanjin groups had lower depression levels than the CG (SMD - 1.44, 95% CI: [-2.46, -0.41], P = .006, I^2^ = 88%) (**Supplementary Figure 3**). The Tai Chi and Baduanjin groups had lower fatigue levels than the CG (SMD - 1.02, 95% CI: [-1.52, -0.53], P <.0001, I^2^=41%) (**Supplementary Figure 4**). The Tai Chi and Baduanjin groups had better sleep quality than the CG (SMD - 1.44, 95% CI: [-2.57, -0.31], P = .01, I2 = 85%) (**Supplementary Figure 5**). The Tai Chi and Baduanjin groups had better quality of life scores than the CG (SMD - 1.44, 95% CI: [5.60, 8.27], P <.00001, I2 = 93%) (**Supplementary Figure 6**).

### 3.5 Subgroup analyses

Analyses of subgroups including shoulder function, anxiety, depression, fatigue, sleep quality, and quality of life scores were performed in accordance with the intervention country (outside China or not) and duration of the intervention (weeks).

#### 3.5.1 Intervention country (China or not in China)

In terms of depression, obvious differences were observed in the SMDs between studies performed in China (70, 90, 102) (P=.002) and studies performed outside of China (96) (P = .67). The Tai Chi and Baduanjin interventions had a significant effect in studies performed in China (SMD = - 1.71, 95% CI: - 2.80 to - 0.62, P = .002) (see **Supplementary Figure 7**). For sleep quality, obvious differences were observed in the SMDs between studies performed in China (82, 102) (P = .01) and studies performed outside of China (96)(P = .49). The Tai Chi and Baduanjin interventions had a significant effect in studies performed in China (SMD = 一1.65, 95% CI: 一2.95 to 一0.35, P = .01) (see **Supplementary Figure 8**). For quality of life, obvious differences were observed in the SMDs between studies performed in China (70, 82, 91, 92, 94, 95, 99) (P <.00001) and studies performed outside of China (94, 97) (P = .23). The Tai Chi and Baduanjin interventions had a significant effect in studies performed in China (SMD = 7.51, 95% CI: 6.09 to 8.93, P <.00001) (**Supplementary Figure 9**).

#### 3.5.2 Intervention duration (weeks)

In terms of shoulder function, 4 out of the 5 studies indicated a significant effect (91, 92, 94, 95) with an intervention period of ≥10 weeks [SMD = 7.63, 95% CI: (6.59 to 8.67), P <.0001]. For the remaining study (100), the pooled effect was SMD = 1.40 (95% CI: −3.31, 6.11; P = .56) within the intervention period. In contrast to the control treatment, Tai Chi and Baduanjin had different effects on shoulder function based on the intervention duration. There was a significant difference in shoulder function in interventions that lasted for longer than 10 weeks (**Supplementary Figure 10**).

Among the 4 studies on anxiety indicators, 1 study (93) with an intervention duration > 12 weeks reported a pooled effect of SMD = −4.64 [95% CI: (−6.59 to −2.69)], P <.00001]. For 3 studies (70, 89, 90) in which the intervention duration was ≤ 12 weeks, the pooled effect was SMD = −1.51 [95% CI (−2.577, −0.45), P = .005]. An intervention period of more than 8 weeks was found to have an obvious effect on reducing breast cancer patients’ levels of anxiety (**Supplementary Figure 11**).

Of the 4 studies regarding depression indicators, 3 studies (70, 90, 96) had an intervention duration of > 8 weeks and reported a pooled effect of SMD = −1.53 [95% CI: (−2.59 to −0.47), P = .005]. For 1 study (102) in which the intervention cycle was ≤ 8 weeks, the pooled effect was SMD = −0.20 [95% CI (−3.98, 3.58), P = .006]. An intervention period of more than 8 weeks was found to have an obvious effect on reducing breast cancer patients’ levels of depression (**Supplementary Figure 12**).

Among the 4 studies reporting fatigue indicators, 3 (70, 96, 98) had an intervention duration > 8 weeks and reported a pooled effect of SMD = −1.30 [95% CI: (−1.88 to −0.73), P <.00001]. For 1 study (102) in which the intervention cycle was ≤ 8 weeks, the pooled effect was SMD = −0.20 [95% CI (−1.19, 0.79), P = .69]. An intervention period of more than 8 weeks was found to have an obvious effect on reducing breast cancer patients’ levels of fatigue (**Supplementary Figure 13**).

Among the 3 studies regarding sleep quality indicators, 2 (82, 96) had an intervention duration > 8 weeks and reported a pooled effect of SMD = −2.88 [95% CI: (−4.39 to −1.38), P = .0002]. For 1 study (102) in which the intervention cycle was ≤ 8 weeks, the pooled effect was SMD = 0.40 [95% CI (−1.30, 2.10), P = .65]. An intervention period of more than 8 weeks was found to have increase sleep quality among breast cancer patients (**Supplementary Figure 14**).

Among the 9 studies reporting quality of life indicators, 1 (150) had an intervention duration > 12 weeks and had a pooled effect of SMD = −7.78 [95% CI: (−17.89 to 2.33), P = .13]. For 8 studies (70, 82, 92, 94, 95, 97–99) in which the intervention cycle was ≤ 12 weeks, the pooled effect SMD = 7.20 [95% CI (5.85, 8.54), P <.00001]. An intervention period of more than 8 weeks was found to have an obvious effect on increasing quality of life among breast cancer patients (**Supplementary Figure 15**).

### 3.6 Sensitivity analysis

In the sensitivity analysis (**Table 3**), the study of Li et al. (90) was excluded, and we observed an obvious change in heterogeneity, which decreased from 91% to 0%. Similarly, when excluding the study by Liao et al. (2022) (82), the heterogeneity level decreased to 0%. Based on these observations, we hypothesize that the outcomes of depression and sleep quality indicators may be the primary sources of heterogeneity in this study. Other potential reasons for heterogeneity include differences in patient populations, inconsistencies in clinical indicators between domestic and overseas studies, and specific treatment methods. The patient characteristics, specific treatment modalities, and applied clinical indicators across the literature are as follows: Li et al. (90), Shanxi (China), aged 37-57 years, breast cancer patients, Baduanjin intervention, shoulder function, anxiety, depression; Liao et al. (2022) (82), Guangzhou (China), aged 46-60 years, breast cancer patients, Baduanjin intervention, sleep quality, and quality of life. The factors mentioned above are all potential sources of heterogeneity in this study.

**Table 3:** Sensitivity analysis for depression and sleep quality indicators.

After excluding the reference	The Result of Heterogeneity:			
	Chi²	df	P	I²
Chen et al., 2013 (102) CES-D	9.03	2	=0.01	78%
Larkey et al., 2015 (96) BDI	7.40	2	=0.02	73%
Li et al., 2017 (90) SDS	1.81	2	=0.40	0%
Wei et al., 2022 (70) HADS	9.48	2	=0.009	79%
Chen et al., 2013 (102) PSQI	5.75	1	=0.02	83%
Larkey et al., 2015 (96) PSQI	13.35	1	=0.0003	93%
Liao et al., 2022 (82) PSQI	0.69	1	=0.41	0%

Sensitivity analysis for depression.

### 3.7 Publication bias

We conducted a thorough analysis of the funnel plots for cognitive function (**Supplementary Figure 13**), shoulder function (**Supplementary Figure 14**), anxiety (**Supplementary Figure 15**), depression (**Supplementary Figure 16**), fatigue (**Supplementary Figure 17**), sleep quality (**Supplementary Figure 18**), and quality of life (**Supplementary Figure 19**). The plots were found to be symmetrical, strongly suggesting the absence of publication bias. Furthermore, the P values exceeded.04, indicating a lack of significant publication bias. Additionally, Egger’s regression test (P=.018) and Begg’s test (P=.02) confirmed the absence of publication bias. All in all, it seems challenging to detect, let alone define, publication bias in the present context because the study conditions and designs appear relatively heterogeneous.

## 4 Discussion

### 4.1 Principal findings

This study systematically evaluated the efficacy of Tai Chi and Baduanjin in breast cancer patients, drawing upon data from 16 studies with a total of 1247 participants. Our findings revealed that Tai Chi and Baduanjin significantly improved cognitive function (SMD = 1.00), shoulder function scores (SMD = 7.34), sleep quality (SMD = -1.44) and quality of life (SMD = 6.94) and reduced depression (SMD = -1.44), anxiety (SMD = -2.22) and fatigue scores (SMD = -1.02). These findings suggest that Tai Chi and Baduanjin are highly effective alternatives for treating breast cancer patients. However, caution is advised in interpreting these results because of the statistical heterogeneity mentioned in the research. To investigate the impact of individual studies on overall risk, sensitivity analysis was conducted to identify the main sources of heterogeneity. Notably, there were large variations in sample size (range: 11–100), intervention type, intervention duration (range: 5–24 weeks), weekly intervention hours (range: 20–90 min/week), and type of control group. (e.g., regular intervention, routine rehabilitation training, waiting list, sham qigong, usual care, no intervention). We acknowledge that cultural background, measurement instruments, and other confounding factors may have contributed to the heterogeneity observed in this systematic review. To the best of our knowledge, this is the first meta-analysis and systematic review to evaluate the effectiveness of Tai Chi and Baduanjin therapy on seven outcome indicators in breast cancer patients. In our study, Tai Chi and Baduanjin therapy significantly improved cognitive function and shoulder function and alleviated depression, anxiety, fatigue and quality of life symptoms.

### 4.2 comparison with previous work

Previous studies have explored the effects of Tai Chi and Baduanjin in cognitive ability (69, 70), alleviating shoulder function impairment (55, 59) and improving mental health problems (72, 73) in breast cancer patients. However, previous studies have reported inconsistent findings regarding the effects of Tai Chi and Baduanjin therapy on improving cognitive function (70), shoulder function (71), reducing falls (75), improving stress and mental health problems (81), improving fatigue, sleeping quality, depression symptom (70) in breast cancer patients. To the best of our knowledge, this systematic review included recent literature (2013-2023) and is the first systematic review and meta-analysis to evaluate examined the effect of Tai Chi and Baduanjin of seven outcome indicators in breast cancer patients. In our study, Tai Chi and Baduanjin were found to significantly alleviate anxiety, depression, and sleep problems and improve cognitive impairment, shoulder function impairment, fatigue, and quality of life (QoL). This systematic review also divided subgroup analysis of the weeks and continental plates, which was not available in the previous systematic review.

### 4.3 Limitations

This study has some limitations that should not be ignored. First, given the nature of systematic reviews, a clear causal relationship cannot be established, as Tai Chi can but only play a supportive role that is preventive and somewhat curative but always in conjunction with other therapeutic methods. Additionally, stress outcome indicators had some impact on the psychological health of breast cancer patients. However, as the only study reported these indicators, a comprehensive comparison could not be made; thus, these indicators were not included in this review. Third, due to the design restrictions in the study, the randomization and blinding methods of the included studies were seldom described in detail. Only five (82, 91, 97, 98, 101) studies were found to detail the randomization method. In contrast, randomization was only mentioned in other studies, and no explanation of the method used was provided. Among them, double-blinding was only implemented in three (96, 97, 101) studies.

### 4.4 Implications

Complementary therapies, particularly mind-body practice, are interventions with great effectiveness for managing treatment side effects and breast cancer symptoms and side effects of complementary therapies (113). Tai Chi (114) and Baduanjin (115) are therapies that complement each other and integrate physical and psychological components. These ancient Chinese mind-body exercises combine breathing exercises, meditation, relaxation techniques, and physical movement (116). Tai Chi and Qigong are two of the most popular traditional aerobic exercises used to treat breast cancer (42). The most common cancers diagnosed among Chinese females was breast cancer, with an, age-standardized incidence rate(ASIR) of 39.1 per 100,000 (117). Of all countries, China faces the largest economic cost of cancers at INT $6.1 trillion, followed by the US (INT $5.3 trillion) and India (INT $1.4 trillion) (118). Our findings have also found that Tai Chi and Baduanjin can have a positive impact on increasing in cognitive ability, shoulder joint function, quality of life, sleep quality and decreasing in anxiety, depression, fatigue symptom among breast cancer patients. With this in mind, we suggest that nursing home caregivers, social workers, and psychologists consider using Tai Chi and Baduanjin in their work, and collaborate with Tai Chi and Baduanjin professionals, it will both physical and psychological health improvements to the breast cancer patients and reduce the financial burden on our country at the same time.

## 5 Outlook

In the future, more research is needed to improve participant motivation, reduce dropout rates, and sustain the benefits of Tai Chi and Baduanjin therapy. Given the study limitations, subgroup analyses for intervention types were not feasible. Therefore, it is essential to conduct in depth, stratified comparisons and discussions of different types of interventions. To validate the effectiveness of Tai Chi and Baduanjin therapy for breast cancer patients, researchers should conduct high-quality studies with large sample sizes. All in all, we should recognize that this research is good to stimulate future research on the many possibilities of Tai Chi and its potential therapeutic benefits as a holistic and preventive approach to a healthy lifestyle, as compared to the more analytical and curative focus in Western discussions. And the take-home message is that Tai Chi is a form of mental relaxation capable of restoring attention and inner resources (25), which is movement-oriented, as it focuses on forms of gentle motion particularly beneficial for older individuals or patients aiding the healing process. Finally, it is a social act that promotes the feeling of togetherness by bringing people together in the true sense of the word (39).

## 6 Conclusion

Overall, Tai Chi and Baduanjin therapy may be associated with an increase in quality of life among breast cancer patients. However, a definitive conclusion could not be reached on the safety and effectiveness of Tai Chi and Baduanjin therapy for improving cognitive ability, shoulder joint abilities, and mental health among breast cancer patients. The overall quality of evidence across all meta-analyses was found to be very low. This was primarily due to concerns about the overall bias within most included studies, high evidence heterogeneity, and reported effect size imprecision. Given these limitations, psychologists, social workers, psychiatrists, and patients should approach Tai Chi and Baduanjin therapy as complementary treatments rather than as replacements for existing interventions. To more robustly evaluate the effectiveness and safety of Tai Chi and Baduanjin for improving specific cognitive abilities, social well-being, and pain among individuals of different age groups, regardless of cognitive status, further reviews and additional studies are needed. It is essential to assess the impact, safety profile, and long-term effects of this therapy to determine its potential as a sustainable and effective intervention in various settings.

## Data Availability

The original contributions presented in the study are included in the article/**Supplementary Material**. Further inquiries can be directed to the corresponding author.
