# Supplementary material for: The effects of Tai Chi and Baduanjin on breast cancer patients: systematic review and meta-analysis of randomized controlled trials
Source: Front Oncol. 2024 Oct 28;14:1434087. doi: 10.3389/fonc.2024.1434087 (PMC11551136; doi:10.3389/fonc.2024.1434087)

Supplementary Materials

Supplementary Figure 1. Forest plot for shoulder function scores


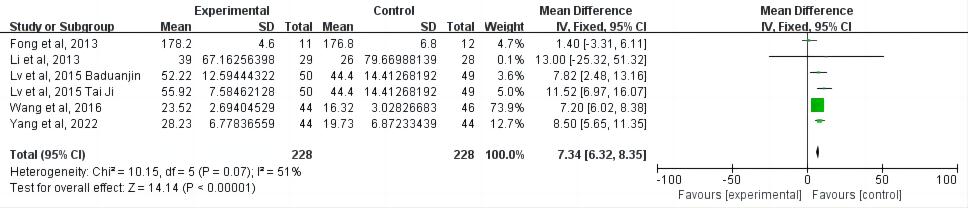


Supplementary Figure 2. Forest plot for anxiety scores


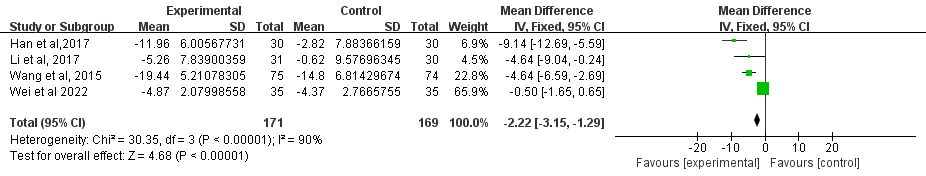


Supplementary Figure 3. Forest plot for depression scores


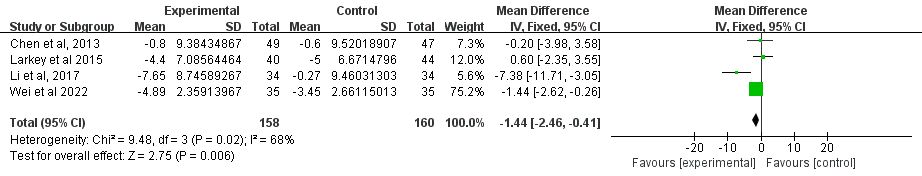


Supplementary Figure 4. Forest plot for fatigue scores


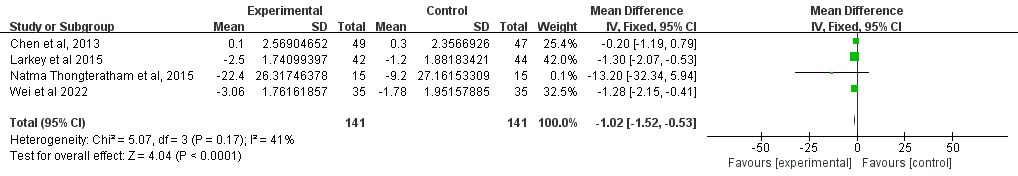


Supplementary Figure 5. Forest plot for sleep quality scores


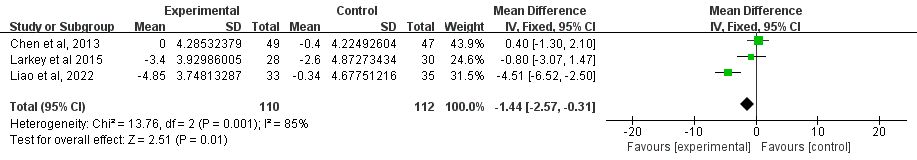


Supplementary Figure 6. Forest plot for quality of life scores


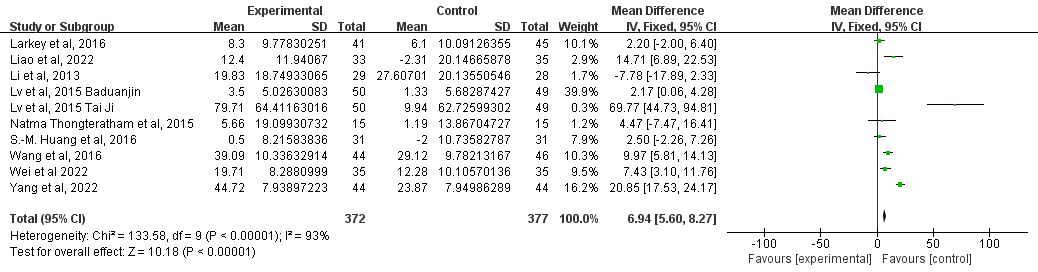


Supplementary Figure 7. Forest graph showing subgroup analysis for sleep quality


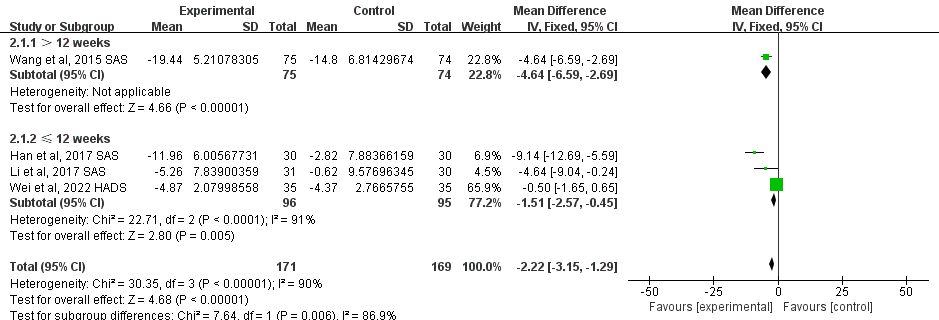


Supplementary Figure 8. Forest graph showing subgroup analysis for quality of life


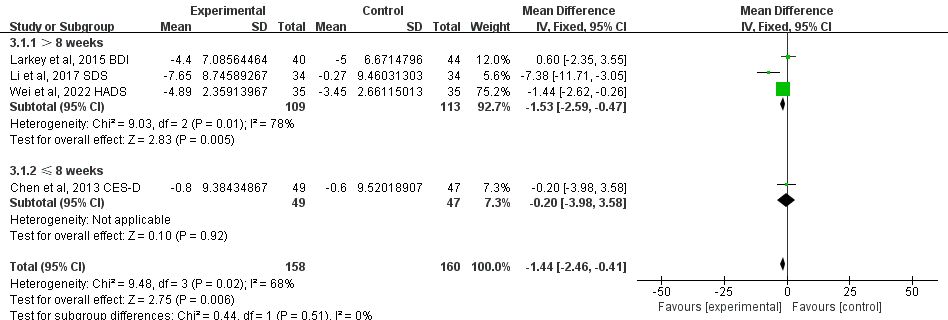


Supplementary Figure 9. Forest graph showing subgroup analysis for shoulder function


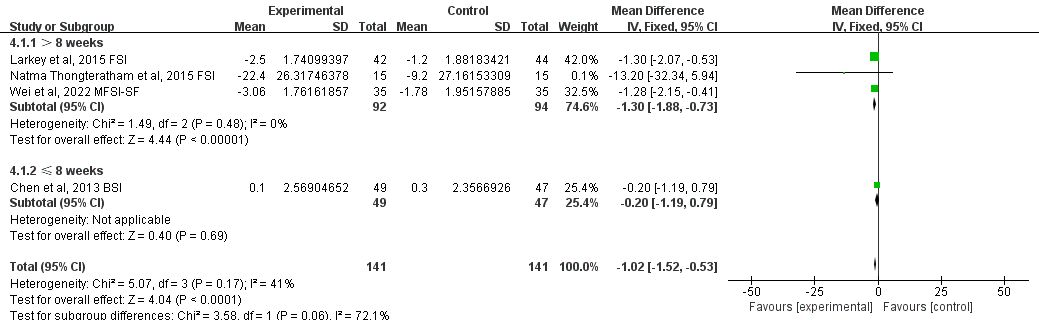


Supplementary Figure 10. Forest graph showing subgroup analysis for anxiety


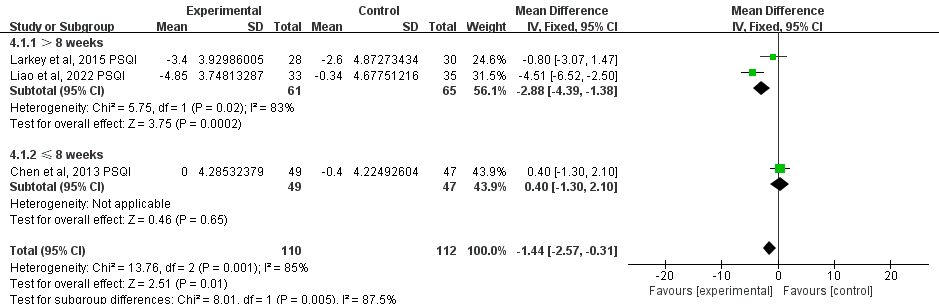


Supplementary Figure 11. Forest graph showing subgroup analysis for depression


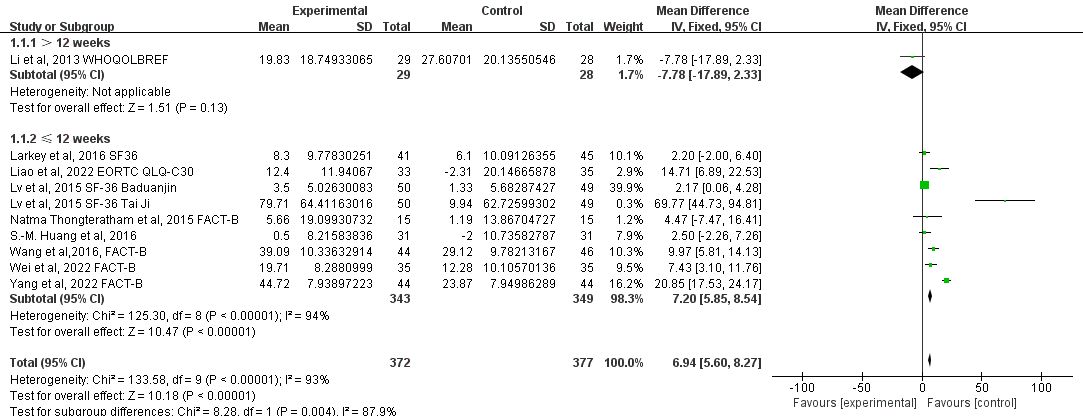


Supplementary Figure 12. Forest graph showing subgroup analysis for fatigue


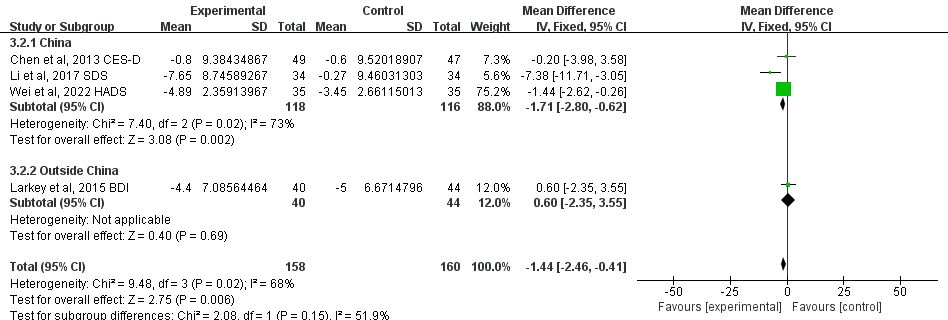


Supplementary Figure 13. Forest graph showing subgroup analysis for sleep quality


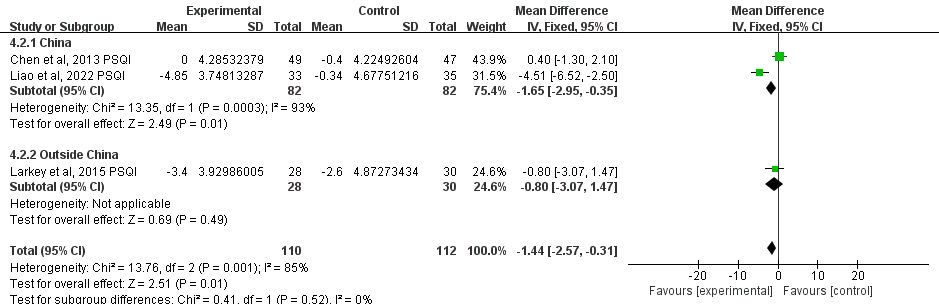


Supplementary Figure 14. Forest graph showing subgroup analysis for quality of life


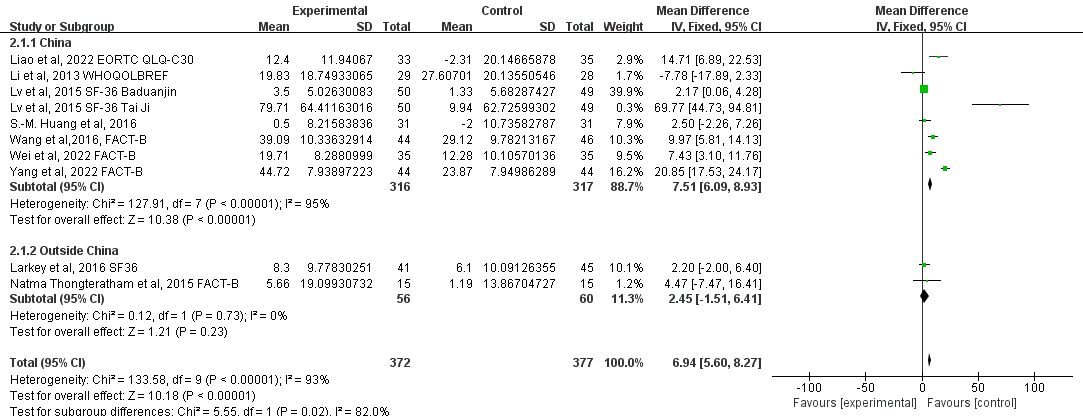


Supplementary Figure 15. Funnel plot for shoulder function


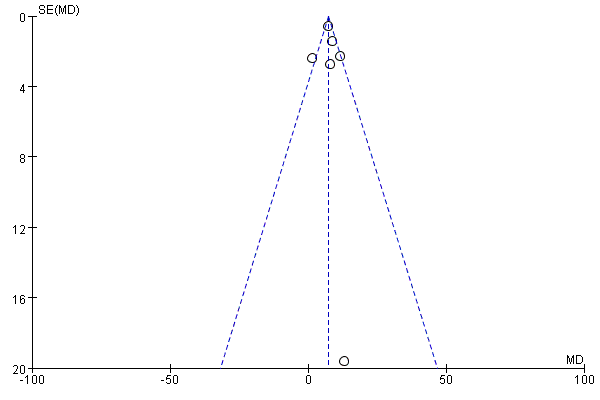


Supplementary Figure 16. Funnel plot for anxiety


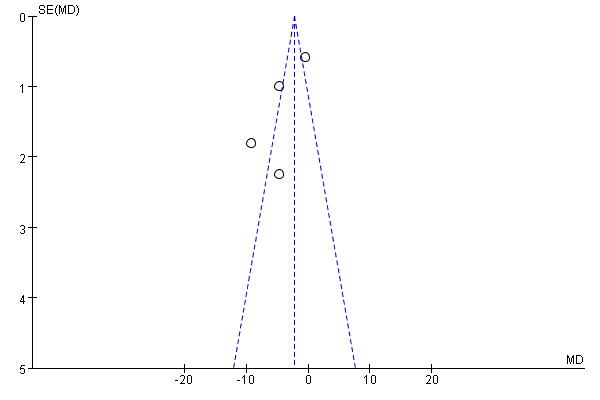


Supplementary Figure 17. Funnel plot for depression


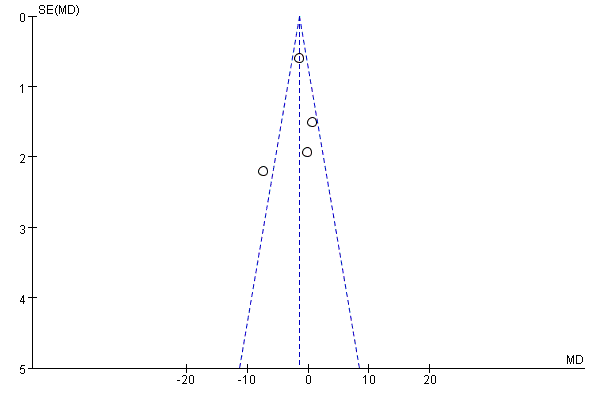


Supplementary Figure 18. Funnel plot for fatigue


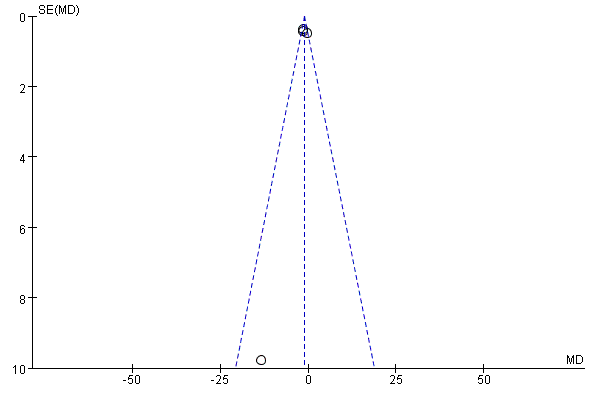


Supplementary Figure 19. Funnel plot for sleep quality


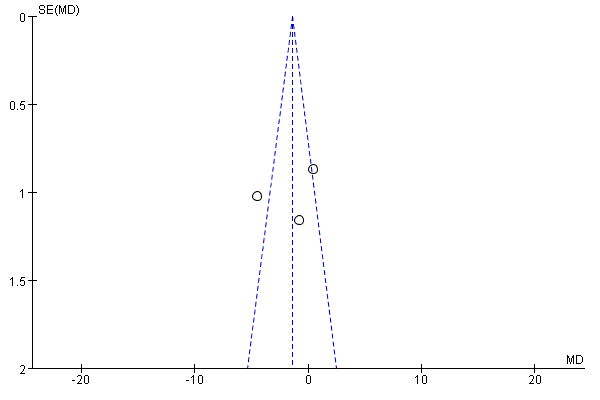


Supplementary Figure 20. Funnel plot for quality of life


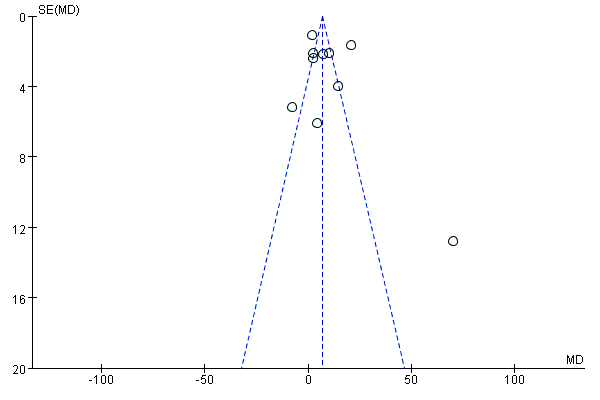

Supplement: Supplementary file 1 [file DataSheet1.docx]
