# Supplementary material for: The effects of Tai Chi and Baduanjin on breast cancer patients: systematic review and meta-analysis of randomized controlled trials
Source: Front Oncol. 2024 Oct 28;14:1434087. doi: 10.3389/fonc.2024.1434087 (PMC11551136; doi:10.3389/fonc.2024.1434087)
Supplement: Supplementary file 2 [file DataSheet2.docx]

Multimedia Appendix 1. PICOS-based eligibility criteria (participation, intervention, comparison, outcomes, and study design)

PICOS Criteria

| Participation | Breast cancer patients |
| --- | --- |
| Intervention | Tai Chi and Baduanjin |
| Comparison | Tai Chi and Baduanjin group and control group |
| Outcome | Cognitive function, Shoulder joint function, Anxiety, Depression, Fatigue, Sleep quality, Quality of life |
| Study design | Randomized controlled trial |
