# Supplementary material for: The effects of Tai Chi and Baduanjin on breast cancer patients: systematic review and meta-analysis of randomized controlled trials
Source: Front Oncol. 2024 Oct 28;14:1434087. doi: 10.3389/fonc.2024.1434087 (PMC11551136; doi:10.3389/fonc.2024.1434087)
Supplement: Supplementary file 3 [file DataSheet3.docx]

Multimedia Appendix 2. PubMed retrieval strategy

| **Multimedia Appendix 2 \| PubMed retrieval strategy.**  #1 (“Tai Chi ”(All Fields) OR “Baduanjin”(All Fields)  #2 “Cognitive Function”(Title/Abstract) OR “Shoulder Joint Function”(Title/Abstract) OR “Mental Health”(Title/Abstract) OR “Anxiety”(Title/Abstract) OR “Depression”(Title/Abstract) OR “Fatigue”(Title/Abstract) OR “Sleep Quality”(Title/Abstract) OR “Quality of LIfe”(Title/Abstract)  #3 #1 AND #2  #4 “Breast Cancer Patients”(Title/Abstract) OR “Breast Cancer Women”(Title/Abstract)  #5 “A pilot study”(Title/Abstract) OR “Randomized Controlled Trial”(Title/Abstract) OR “RCT”(Title/Abstract)  #6 #3 AND #4 AND #5 |
| --- |
